# Testing reference genes for transcript profiling in *Uromyces appendiculatus* during urediospore infection of common bean

**DOI:** 10.1371/journal.pone.0237273

**Published:** 2020-08-06

**Authors:** Tobias I. Link

**Affiliations:** Department of Phytopathology, Institute of Phytomedicine, Faculty of Agricultural Sciences, University of Hohenheim, Stuttgart, Germany; University of Nebraska-Lincoln, UNITED STATES

## Abstract

*Uromyces appendiculatus* is a major pathogen on common bean. Like other rust fungi, it uses effectors to influence its host plant. Effectors are assumed to possess characteristic expression profiles, reflecting their activity during the infection process. In order to determine expression profiles using RT-qPCR, stably expressed reference genes are necessary for normalization. These reference genes need to be tested. Using samples representing seven different developmental stages of the urediospore-based infection process we employed RT-qPCR to measure the expression of 14 candidate reference genes and determined the most suitable ones based on the range of Cq values and comparative calculations using the geNorm and NormFinder algorithms. Among the tested genes *RPS14* had the smallest Cq range, followed by *Elf1a* and *Elf3*; geNorm rated *Tub* and *UbcE2* best with *CytB* as a third and NormFinder found *UbcE2*, *Tub* and *Elf3* as best reference genes. Combining these findings using equal weight for the rankings *UbcE2*, *Elf3* and *Tub* can be considered the best reference genes. A combination of either two reference genes, *UbcE2* and *Tub* or three reference genes, *UbcE2*, *Tub*, and *Elf3* is recommended for normalization. However, differences between most genes were relatively small, so all tested genes can be considered suitable for normalization with the exception of *RPS9*, *SDH*, *Ubc* and *PDK*.

## Introduction

*Uromyces appendiculatus* is a major pathogen on common bean, *Phaseolus vulgaris* [1]. Common bean is cultivated almost globally both in subtropical and temperate regions. Both pods of *P*. *vulgaris* and dry seeds are consumed. With a high protein content and also rich in minerals like iron and zinc common bean is a highly important component of the diet of many people, especially in developing countries where meat may not be easily available [2].

*U*. *appendiculatus* is an autoecious rust fungus forming all phases of its infection cycle on one host. This means that in spring basidiospores are formed from teliospores on bean leaf litter, these can infect young bean plants to form pycnia and aecia and aeciospores and later urediospores will further infect the plants. Especially urediospores are produced in vast amounts and can lead to epidemic spread of the disease. Later in the year teliospores are formed to complete the sexual cycle and for overwintering. The urediospore stage of the infection cycle is of highest interest since this is the stage that is most frequently encountered in the field and leads to the highest yield losses. Also subtropical races of *U*. *appendiculatus* exist that have lost the ability to form teliospores [1].

*U*. *appendiculatus* can be effectively controlled with fungicides and also cultural practices like intercropping or crop rotation together with good sanitation in the field can reduce disease incidence. Since common bean is mostly produced by small scale farmers the efficiency of fungicide application is very limited. Therefore, breeding for resistance is highly important. Several resistance genes have been identified and both cultivar mixes and multi-lines are used for rust control. However, due to the high genetic variability of the pathogen the efficiency of these efforts remains limited [1]. Therefore, broad spectrum and stable resistance is still a highly important objective. Here, studies on rust biology, genetics and physiology can contribute to identify the optimal strategy to establish durable resistance. Studying effectors of rust fungi and their targets is a novel strategy to identify novel sources for resistance. This strategy is also pursued for *U*. *appendiculatus* [3–6]. Part of the characterization of (candidate) effectors is to determine their expression profile. The gold standard for expression profiling is RT-qPCR. For normalization in RT-qPCR internal reference genes are needed. Optimally reference genes should be tested for their stability under the studied conditions [7–9]. Optimal reference genes can be identified by comparing between a number of candidate reference genes, for example housekeeping genes. Algorithms that can identify the most stable genes from these comparisons have been published [10, 11]. While reference genes for *P*. *vulgaris* have been validated, including biotic stress conditions [12], no systematic test for the optimal reference genes has been performed for *U*. *appendiculatus*, so far. This might also explain why Cooper et al. [13] used a plant gene as reference for measuring gene expression and fungal biomass in their gene silencing study. Here, a study to identify suitable reference genes for normalization in *U*. *appendiculatus* during the phases of infection is presented.

The urediospore infection can be structured into three developmental phases: the penetration phase, the parasitic phase and a sporulation phase [14]. For penetration the spore first attaches firmly to the leaf. Then germination occurs, leading to the formation of germ tubes (gt) and later appressoria (ap) that are positioned over stomata. A penetration hypha and the so called substomatal vesicle are formed, followed by an infection hypha and the haustorial mother cell. The haustorial mother cell is formed upon contact with a plant mesophyll cell and is the basis for plant cell penetration and, as the name implies, for haustorium formation. Up to the haustorial mother cell these infection structures can be generated *in vitro* on polyethylene sheets that were treated to produce grooves resembling the edges of stomata. Formation of haustoria marks the beginning of the parasitic phase that lasts for several (five to nine) days before sporulation starts. During the parasitic phase the fungus spreads through the plant tissue forming more haustoria. Then hyphae form aggregates that differentiate into uredia producing urediospores. Sporulation can go on for a long time provided the host plant stays fit. However, leaves that are strongly infected—which is always the case upon artificial inoculation—become senescent after a few days of sporulation. For this study samples were evaluated that represent the three phases of the urediospore infection.

## Materials and methods

### Plant material

Common bean (*P*. *vulgaris*) cultivar Primel (Vilmorin, La Ménitré, France) was grown in the greenhouse with a 16 h light / 8 h dark regime at 22 °C / 18 °C. The plants were inoculated when they were 21 d old; at this stage they were forming the second trifoliate leaf.

### Fungal isolate, plant inoculation, and *in vitro* production of early infection structures

Urediospores of *U*. *appendiculatus* (SWBR1, laboratory collection, Phytopathology, University of Hohenheim, Stuttgart, Germany) were suspended in water (0.05%) together with 0.08% milk powder and 0.01% Tween22 (w/v) and stirred for 30 min at room temperature in the dark. This suspension was sprayed onto *P*. *vulgaris* plants using a chromatography vaporizer. The plants were then kept at 95% humidity in the dark at 15 °C for 24 h and then put back to the greenhouse with 16 h light / 8 h dark at 15 °C / 13 °C.

To obtain germ tubes (gt) and appressoria (ap), urediospores were finely dispersed onto polyethylene (PE) sheets using fine gauze material for sifting. For producing ap, the PE sheets were scratched using a brass brush beforehand. After dispersing the spores, the PE sheets were sprayed with water using a chromatography vaporizer and then kept at 20 °C and 95% humidity in the dark. For gt generation the structures were harvested after 4 h, for ap after 9 h. Formation of both gt and ap was checked by microscopy. For later RNA preparation the structures were scraped from the PE sheets, dried by vacuum filtration and stored in 2 ml tubes at -70 °C after freezing in liquid nitrogen.

### RNA preparation and cDNA synthesis

Harvesting of structures grown *in vitro* is described above. Tissue from soybean leaves either uninfected or infected at different stages was gained by cutting out pieces with a cork borer. A cork borer with 12 mm diameter was used; every sample was composed of four pieces cut out from different leaves giving an amount of roughly 100 mg. The samples were collected in plastic tubes, frozen in liquid nitrogen and kept at -70 °C until RNA preparation.

While still frozen, samples were homogenized with a FastPrep^®^-24 MP (Biomedicals GmbH, Eschwege, Germany) set to a shaking speed of 4.5 m/s. For this, two 2 mm steel beads were added to the sample and homogenization was run three times for 20 s; in between homogenization rounds the samples were cooled again in liquid nitrogen. The Agilent Plant RNA Isolation Mini Kit (Agilent Technologies, Santa Clara, CA, USA) was used for RNA preparation. The preparation was done according to the manufacturer’s instructions. Because the mini prefiltration columns tended to clog otherwise, an additional centrifugation step to remove cell debris was added after adding the extraction buffer. RNA quality and concentration were checked photometrically by measuring OD_260_ and the OD_260_/OD_280_ ratio. To test for RNA integrity, also DNA electrophoresis was performed with 1% agarose gels under denaturing conditions. The isolated RNA samples were stored at -70 °C.

To remove residual DNA RNA was treated with DNaseI (Thermo Fischer Scientific, Waltham, MA). Following the recommendations of the manufacturer, 1 μg of total RNA was treated in a 10 μl reaction. cDNA synthesis was performed with the RevertAid RT Reverse Transcription Kit (Thermo Fischer Scientific), also following the manual. To the 11 μl RNA resulting from DNAseI treatment after addition of EDTA the reverse transcriptase and corresponding buffers were added together with random hexamer primers to give a 20 μl reaction mix that was incubated at 25 °C for 10 min, 42 °C for 60 min, and heat inactivated for 10 min at 70 °C. For production of larger amounts of cDNA, reactions were scaled up by proportionally increasing all amounts and volumes. Storage of the cDNA was at -20 °C.

### Real time PCR parameters

All Real Time PCR reactions were run on a CFX96^™^ Real-Time-PCR System (Bio-Rad Laboratories, Hercules, CA, USA). FrameStar^®^ 96 Well Skirted PCR Plates with white wells and black frame (4ti-0961, 4titude, Brooks Automation, Chelmsford, MA, USA) were used. Plate design, data logging, and basic data analysis including the determination of Cq values as well as the calculation of primer efficiencies from dilution series was performed with the Bio-Rad CFX Manager 2.1 (Bio-Rad Laboratories). For Cq determination, thresholds were first used as determined automatically by the CFX Manager, for full comparability 3000 rfu as a rounded estimated average of automatic thresholds was used.

20 μl Real Time PCR reactions were performed using the SensiFAST^™^ No-ROX mix (Bioline Reagents Ltd., London, UK) with primers at a concentration of 0.4 μM and 2 μl cDNA template (directly from reactions described above) or alternatively water in the NTC or RNA (1 μg / 20 μl in the same buffers as the cDNA) in the NRT controls respectively.

A two step PCR protocol was used with an initial denaturation step of 95 °C for 5 min, followed by 40 cycles with 5 s at 95 °C and 15 s at 60 °C for annealing and elongation. A melt curve analysis was also run starting at 65 °C to 95 °C in steps of 0.5 °C with a duration of 5 s and measuring of fluorescence after every step. For correct readout, the instrument was set to the BR white plate setting and the SYBR/FAM only scan mode was used.

### Data analysis

For determination of the most stable candidate reference genes the GenEx software package (GenEx 6.0.1.612, MultiD Analysis AB, Göteborg, Sweden) was used. Cq values exported from the CFX Manager were first averaged for the replicates, and then corrected for efficiency. Both the NormFinder and geNorm algorithms were run as implemented in GenEx. Graphs in this publication showing Cq values are showing Cq values after efficiency correction. The GenEx tool Data editor was also used for normalization and calculation of relative expression data. All graphs were created in MS Excel (Microsoft Corporation, Redmont, WA, USA).

### Primers

Primers (Table 1) were designed using the webtool Primer3 [15, 16] with the following settings: Primer size range 18–23 nt with an optimum of 20 nt, optimal melting temperature (AT+GC) 60 °C with a range from 58–62 °C, GC content 50% with a range from 30–70%, product size 75–150 bp. Other settings were kept at default. The primers were cross-checked with GeneRunner (version 6.5.52 Beta, generunner.net) and occasionally shifted by a few bases to have a G or C at the 3’ end. Primers were ordered in HPCL quality from biomers.net GmbH (Ulm, Germany). Primers were tested for efficiency using cDNA from RNA from spores in four concentrations, undiluted, 1:10, 1:100, 1:1000. NTC and NRT controls were also included and the primers were also tested against cDNA from RNA prepared from non-infected plant material. When primers were found unsuitable due to formation of artifacts (in NTC), alternative primers proposed by Primer3 were used or, as a third alternative, primers were designed directly using GeneRunner.

**Table 1:** Oligonucleotides used in this study.

Gene	Primers	Sequence 5’-3’	Position^a^	Amplicon size	Efficiency [%]
*Act*	UaActf	ACTTGATCTTGCCGGTCGAG	696–715	83	98.2
	UaActr	CGGCAGTGGTGGTAAAGCTA	759–778		
*ASUB*	UaASUBf	TTGCCACCGCCATTCAAAAG	1413–1432	84	95.1
	UaASUBr	CGGAGAGCTCATCCATTCCC	1477–1496		
*CytB*	UaCytbf	CCAGTTTCGATCGTACCGGA	1043–1062	124	98.0
	UaCytbr	CCGGTATGGCTAGCAGGATT	1146–1166		
*Elf1a*	UaElf1af	TCTGTCGCATATCACGGACG	387–406	97	85.9
	UaElf1ar	TGCCTTGTCAAGATGGTCCC	463–482		
*Elf3*	UaElf3f	AACGGCCAAGTCGAAGGATT	1466–1486	138	92.3
	UaElf3r	TTCACGTGTGGCCTGGATAC	1583–1603		
*GAPDH*	UaaGAPDHf2	CCTTCTTGGCACCTCCCTTC	1229–1248	134	101.7
	UaaGAPDHr2	TGATGTCTATGCCGAGCGAG	1343–1362		
*PDH*	UaPDHf	GAGAAGCACCAATGACCCGA	1128–1147	154	102.3
	UaPDHr	TGGATCGGGTTCAGGACTCT	1262–1281		
*PDK*	UaaPDKf2	AAAGGACTGAGCGTACCAATC	1066–1086	152	93.5
	UaaPDKr2	CTGCTGGAACTTTAATGAGGG	1197–1217		
*RPS9*	UaRPS9f	GCGTTGAGGGTTGAGGAAGA	730–750	100	113.9
	UaRPS9r	AAAGAGAGTACCGCCAGAGC	809–829		
*RPS14*	UaaRPS14f2	GGCAGTGATTCCAACCTCC	565–583	126	102.8
	UaaRPS14r2	GAAACCGTCTCCCGTGTCAC	671–690		
*SDH*	UaSDHf	TCGGACATGTCTTGGTGCAA	393–413	150	105.3
	UaSDHr	AATACTTGGGTCCGGCTGTG	522–542		
*Tub*	UaaTubf	AGCACCAAATCCAGACCCAG	1160–1179	159	100.4
	UaaTubr2	GCTGCCAACAACTATGCTCG	1299–1318		
*Ubc*	UaUbcf	CGTTCGGCCATCCTCTAACT	499–519	125	99.9
	UaUbcr	CCTTAGAGGTGGAGTCTTCGG	602–623		
*UbcE2*	UaUbcE2f	CCACCCGAAGGAATCAGAAT	177–196	82	97.3
	UaUbcE2r	GTGTTTCAGCCGGACCCATA	238–257		
*Uaca_9*	Uaca_9_qPCR_2f^b^	GCTCTGTTTTCACTCGTCGC		172	95.6
	Uaca_9_qPCR_2r^b^	AGGGTGCAGTTTGTGGCGG			
*Uaca_12*	Uaca_12_qPCR_2f^b^	TCTCGGTGGTGGTATGAATG		233	81.8
	Uaca_12_qPCR_2r^b^	GTGGTCTGCGATATGGCTTG			

^a^Primer position in the mRNA / cDNA sequence. Sequence accessions are listed in Table 2.

^b^Primers from reference [4].

## Results and discussion

### Infection stages sampled

Samples were taken to represent the major phases of urediospore infection, the penetration phase, the parasitic phase with formation of haustoria and the sporulation phase. During the penetration phase the amount of fungal RNA in the infected leaf is too low for reproducible amplification with RT-qPCR. Therefore, we prepared RNA just from urediospores (sp), from germinated spores or germ tubes (gt) and from infection structures differentiated on structured PE sheets for 9 h when more than 50% of all structures showed appressoria (ap). Incubating for a longer time might have led to a higher percentage of structures reaching the appressorial stage. However, our earlier study on reference genes for *Phakopsora pachyrhizi* [17] showed, that waiting too long also leads to degradation of RNA. Collectively sp, gt, and ap are also named *in vitro* stages or samples as opposed to *in planta* samples.

Infected plants were sampled at 3 days post inoculation (dpi), 5 dpi, 7 dpi, and 14 dpi. 3 dpi and 5 dpi represent the parasitic phase, 7 dpi marks the beginning of the sporulation phase and 14 dpi the later sporulation phase with leaf senescence. 3 dpi was considered the earliest time point when amplification with RT-qPCR from infected plant samples was possible.

### Candidate reference genes

In our study on reference genes for *P*. *pachyrhizi* [17] we tested 15 candidate reference genes carefully chosen from other studies testing or using reference genes for rust fungi [18–21]. Since this study was successful this previous choice was regarded as sensible, useful, and good to be used again. So, for this present study on *U*. *appendiculatus*, it was decided to test the respective homologous genes. The homologs were identified by BLASTing the predicted protein sequences from *P*. *pachyrhizi* against the predicted protein sequences of the *U*. *appendiculatus* transcriptome [3]. This approach allowed the identification of the genes and sequences listed in Table 2. For *RPS11* (40S ribosomal protein S11) no adequate homologous sequence could be identified.

**Table 2:** Candidate reference genes for *U*. *appendiculatus*.

Gene designation	description	GenBank (TSA)Accession / Sequence name
*Act*	actin	GACI01001365 / Ua_contig14123
*ASUB*	ATP synthase β subunit	GACI01005254 / Ua_contig00116
*CytB*	cytochrome b	GACI01000513 / Ua_contig05727
*Elf1a*	elongation factor 1 α	GACI01005361 / Ua_contig00283
*Elf3*	elongation factor 3	GACI01006120 / Ua_contig03597
*GAPDH*	glyceraldehyde-3-phosphate dehydrogenase	GACI01000857 / Ua_contig04879
*PDH*	pyruvate dehydrogenase (e1 component subunit α)	GACI01002393 / Ua_contig05162
*PDK*	pyruvate dehydrogenase kinase	GACI01001817 / Ua_contig06132
*RPS9*	40S ribosomal protein S9	GACI01003329 / Ua_contig10941
*RPS14*	40S ribosomal protein S14	GACI01004410 / Ua_contig01402
*SDH*	succinate dehydrogenase	GACI01004552 / Ua_contig01571
*Tub*	α tubulin	GACI01005215 / Ua_contig00546
*Ubc*	ubiquitin	GACI01005464 / Ua_contig01089
*UbcE2*	ubiquitin conjugated enzyme	GACI01001070 / Ua_contig02159

### Expression of the candidate reference genes in different samples

For the selected genes primers were designed and as recommended by Pfaffl [22] amplification efficiencies were determined (Table 1). For *GAPDH*, *PDK*, *RPS14*, and *Tub*, the primers that were chosen first produced artifacts in the NTC. So for these genes alternative primers were chosen; for *GAPDH* and *RPS14* this was a modified version of alternative primers suggested by Primer3; for *PDK* both primers and for *Tub* the new reverse primer were freely chosen using GeneRunner.

**Fig 1:**
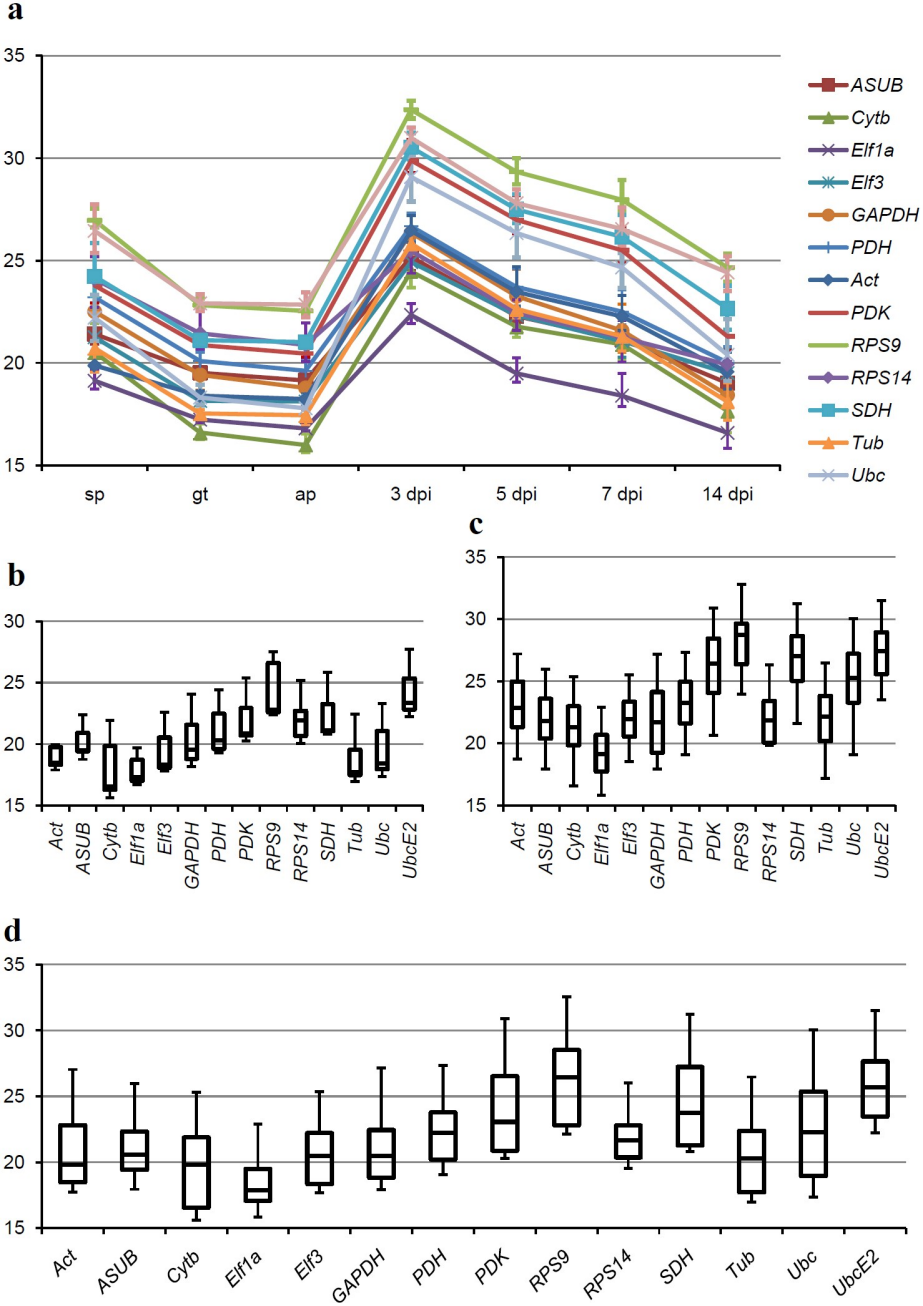
Cq values for the candidate reference genes in all samples. (a) Plot of Cq values of the different samples. For every gene the geometric means of the Cq values over the biological triplicates are shown; error bars represent the highest or lowest values respectively. (b) Box plots of the Cq values from the *in vitro* samples for every gene. (c) Box plots of the Cq values from the *in planta* samples for every gene. (d) Box plots of the Cq values from all samples for every gene. The bars in the box plots indicate the minimum and maximum values and the boxes depict the quartiles. All Cq values for the three biological replicates are represented.

RT-qPCR was run for all candidate reference genes and all samples. For an overall crude impression on expression of the candidate reference genes the efficiency corrected means of the Cq values of the technical replicates were plotted for all samples (Fig 1). In the plot that shows the Cq values for all samples separately (Fig 1a), it initially seems that the expression of all candidate reference genes is not stable between the samples. This apparent instability is not contradictory to the use of these potential reference genes, however. The differences in the Cq values can be explained by general changes in the percentage of fungal mRNA in the total RNA prepared from the different samples: Cq values for the gt samples are generally lower than those for the sp samples. On average the difference between the Cq values for the two stages is three, meaning that gene expression in germinated spores is eight times higher than in the spores. Since the metabolism is resting in the spores, this difference is to be expected. By contrast, the differences between the gt samples and the ap samples are very small, as should be expected for stably expressed genes. This is, however, in contrast to our results for *P*. *pachyrhizi* where Cq values for the appressorial samples were much higher than for the germinated spore samples [17]. As already mentioned, we attributed these higher Cq values to degradation of mRNA in those specimen that were already dead for a while at the time they were collected. The fact that Cq values for the chosen housekeeping genes are now stable between the gt and ap samples is clearly a better and correct result and it can be concluded that the decision to shorten the incubation time for appressoria generation was the right choice. There is a second shift in Cq values from an average of 19.2 in the ap samples to an average of 27 in the 3 dpi samples. The reason for this shift is that in the *in planta* samples the fungal RNA is only a fraction of the total RNA—fungal RNA is “diluted” by the RNA of the host plant. Towards the later stages the average Cq values for the samples are again decreasing. This is explained by the increasing fungal biomass in the leaf over time as the fungus is growing. Since the graphs for the different candidate reference genes are mostly parallel, it appears that these genes nicely represent overall mRNA abundance in the different samples.

Together with all other samples also the samples taken from non-infected plants were tested. Almost all primer pairs gave some amplification with this sample. The Cq values obtained from this control were all >30 (S1 File) and the melting curves showed peaks at similar temperatures as the melting curves obtained during testing the primer efficiencies. This might indicate that the primers also have some affinity to plant RNA, presumably the *P*. *vulgaris* homologs of the candidate reference genes. The possibility that the non-infected plant RNA sample actually contained some *U*. *appendiculatus* contamination might be another reason for these results. However, since the RNA was also tested with other *U*. *appendiculatus* primers with no amplification, this eventuality can be ruled out. Subsequently these Cq values were compared with those for the other samples. Almost all were much higher, so it can be supposed that the products from the amplification of plant RNA do not interfere with the analysis of the amplification of the fungal genes. Two exceptions were noted: *RPS9* and *SDH* that both have relatively high Cq values came close to the Cq values of the control in the 3 dpi sample. Therefore, *RPS9* and *SDH* were excluded from all further analyses including *in planta* samples.

### Identification of the best reference genes

To find the most suitable reference genes the range of Cq values over all samples was considered for all genes. To provide graphical representations of these ranges the Cq values are represented in box plots in Fig 1b to 1d. These box plots represent the Cq ranges for the candidate reference genes either for just the *in vitro* samples (Fig 1b), the *in planta* samples (Fig 1c) or all samples together (Fig 1d). The Cq range was narrowest for *Act* when just the *in vitro* samples were considered (Fig 1b); when the *in planta* samples or all samples were considered *RPS14* had the smallest range of Cq values (Fig 1c and 1d) making it the gene with the least difference in mRNA abundance over all tested samples. *Ubc*, *PDK*, *RPS9*, *SDH*, and *Cytb*, on the other hand, had a very wide range of Cq values. These Cq ranges as a first aspect for deciding, which is the best reference gene, were used for a ranking (Table 3). This ranking was included into an overall ranking to represent the aspect of the Cq range.

**Table 3:** Ranking of the reference genes.

Gene	Ranking by											
	Cq range			geNorm			NormFinder			overall		
	*in vitro*	*in planta*	all stages	*in vitro*	*in planta*	all stages	*in vitro*	*in planta*	all stages	*in vitro*	*in planta*	all stages
***Act***	1	7	8	14	9	9	14	1	8	**11**	**5**	**10**
***ASUB***	2	5	4	13	7	7	11	5	7	**9**	**5**	**6**
***CytB***	14	8	10	10	3	3	12	4	6	**14**	**3**	**7**
***Elf1a***	3	3	2	12	8	8	10	8	9	**7**	**8**	**7**
***Elf3***	4	2	3	1	4	4	5	9	4	**2**	**3**	**2**
***GAPDH***	12	10	6	6	6	6	7	7	5	**7**	**10**	**5**
***PDH***	9	6	5	5	5	5	3	2	3	**5**	**2**	**4**
***PDK***	8	13	13	4	10	10	4	11	10	**4**	**11**	**11**
***RPS9***^a^	7	9	12	11			13			**12**		
***RPS14***	6	1	1	7	11	11	6	10	11	**6**	**9**	**9**
***SDH***^a^	5	12	11	1			1			**1**		
***Tub***	10	11	9	3	1	1	2	6	22	**3**	**7**	**3**
***Ubc***	13	14	14	9	12	12	9	12	12	**12**	**12**	**12**
***UbcE2***	11	4	7	8	1	1	8	3	1	**10**	**1**	**1**

All numbers indicate ranks. The genes were ranked in three ways; first by Cq range with narrowest range best and widest range last and by the algorithms geNorm and NormFinder. All rankings were performed either separately for the *in vitro* and the *in planta* samples or for all samples together. The overall ranking is based on the averages of the three other rankings.

^a^*RPS9* and *SDH* were excluded from all rankings (except Cq range) that include *in planta* samples.

Next, the stability of expression was tested with the algorithms geNorm and NormFinder. Both algorithms calculate differences between the Cq values of the different genes and compare these differences between the different samples provided. When these differences are mostly constant between the different samples the gene expression is deemed stable, the more variations in the differences are noted, the less stable is the expression of the gene. While geNorm repeatedly compares each gene against an average of all other genes and removes the gene with the worst performance (the highest M value) from the group after every round of calculations, the NormFinder algorithm is model based [11] and as implemented in GenEx provides a standard deviation (SD) for every gene. The functioning of geNorm determines that there is always a pair of genes performing best and the authors state that at least three genes should be used for normalization [10]. NormFinder, on the other hand, can rank all genes by SD and also provides an accumulated standard deviation for a group of genes that indicates what should be the optimal number of genes used for normalization. Like for the Cq ranges these analyses were performed both separately for the *in vitro* samples and the *in planta* samples and for all samples together.

**Fig 2:**
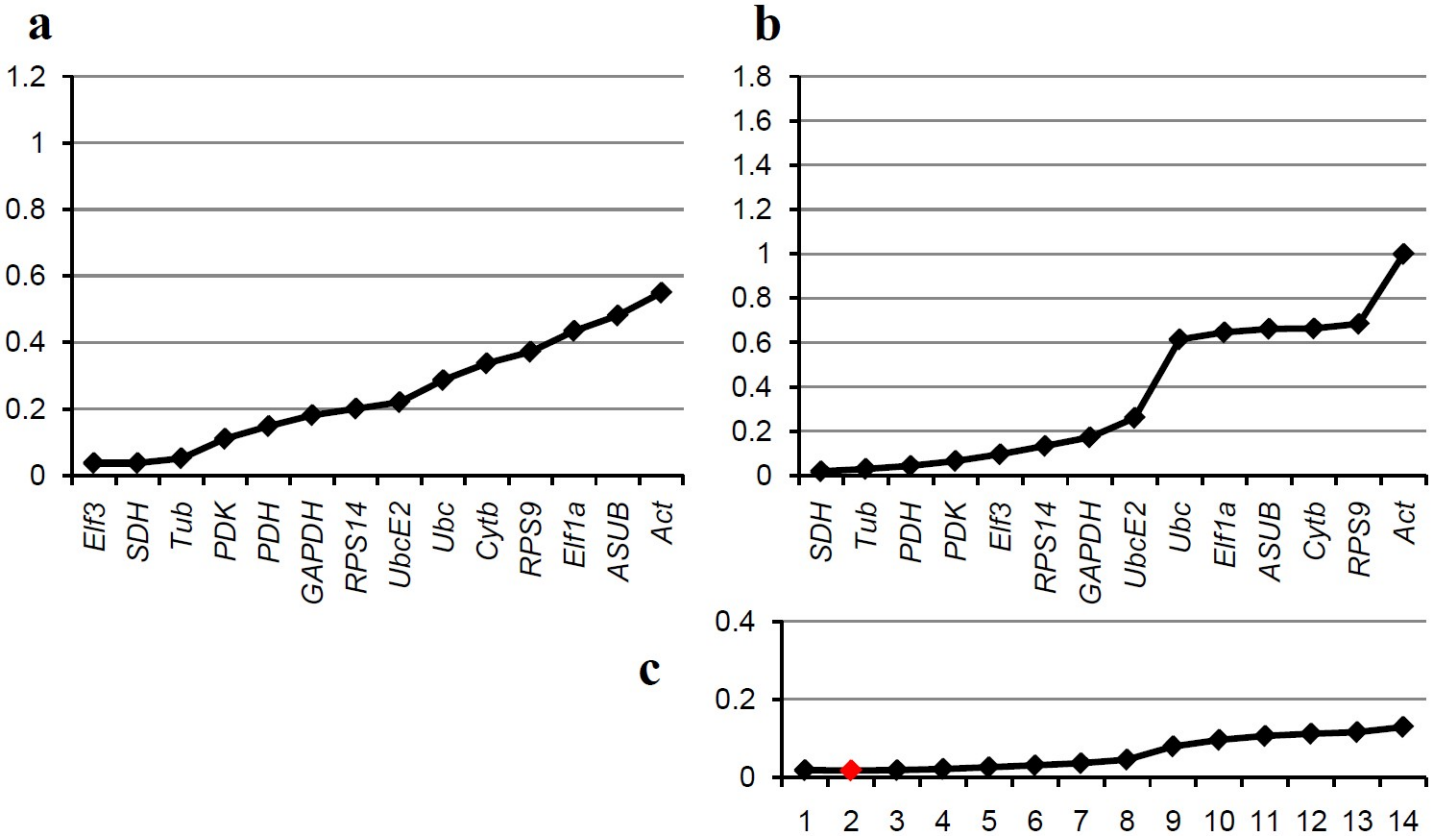
Comparative analysis of the candidate reference genes for *in vitro* samples. Genes are ranked based on the results of the algorithms geNorm and NormFinder, best genes left. (a) Ranking by the geNorm algorithm; the graph shows the M-values for the different genes. (b) Ranking by the NormFinder algorithm; the graph shows the standard deviations (SD) for the different genes. (c) Accumulated standard deviations by NormFinder for different numbers of genes; the optimum is shown by a red indicator.

Fig 2 shows the results for the *in vitro* samples. The geNorm algorithm predicts *Elf3* and *SDH* as the best genes with *Tub* third (Fig 2a), while NormFinder finds *SDH* and *Tub* best with *PDH* third (Fig 2b). Altogether all values are quite low so most of the genes seem to be suitable although there is a group of seven genes including *SDH*, *Tub*, *PDH*, *PDK*, *Elf3*, *RPS14*, *GAPDH*, and *UbcE2* that are markedly better than the other six. These two groups are the same for both algorithms indicating an overall similarity of the results from both algorithms. From accumulated SD it appears that two reference genes should be optimal (Fig 2c) but there is so little difference between the values that even one gene could be sufficient. Based on these results, either *SDH*, *Tub*, or *Elf3* could be used as reference genes for normalization between *in vitro* samples.

**Fig 3:**
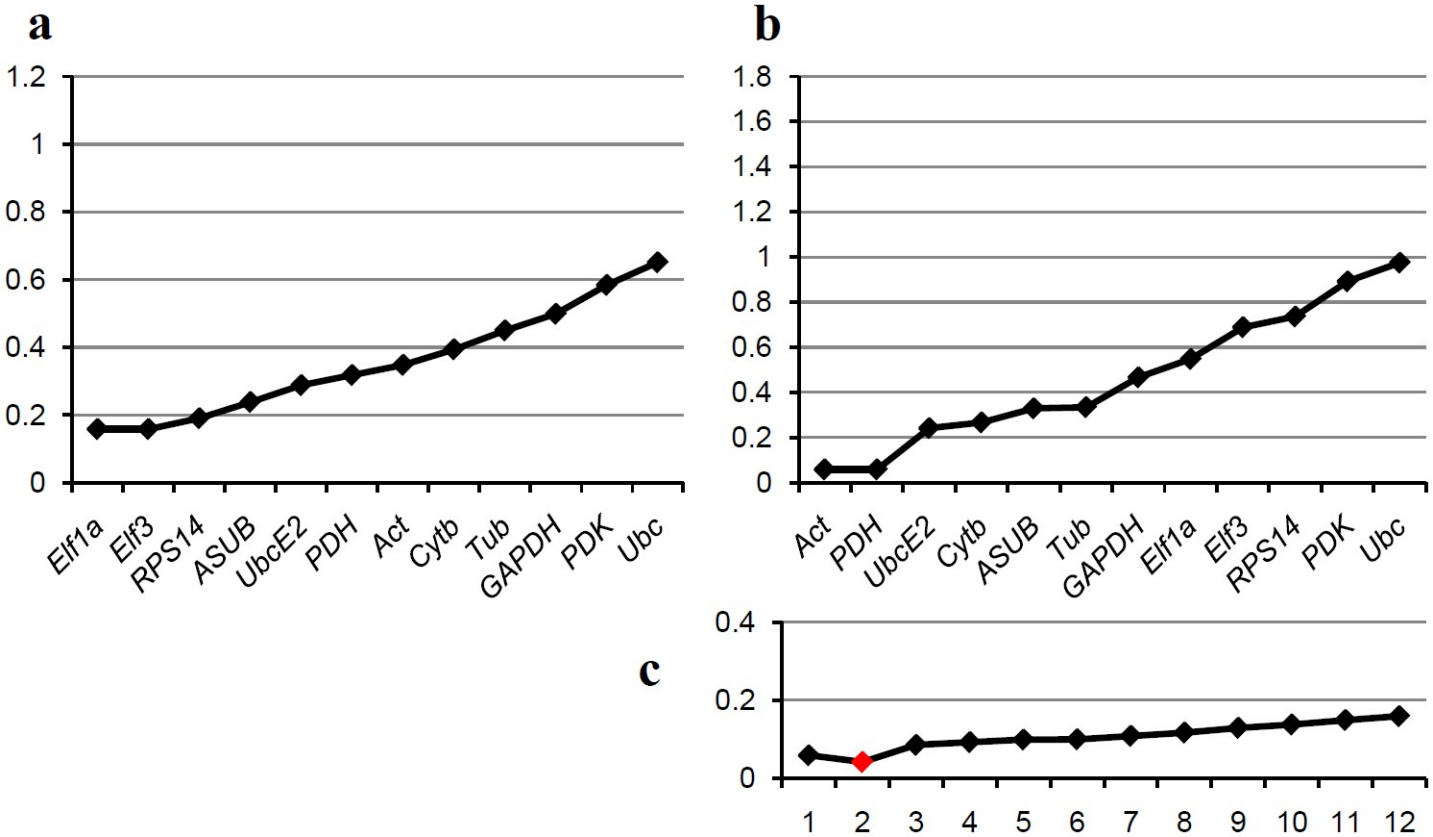
Comparative analysis of the candidate reference genes for *in planta* samples. Genes are ranked based on the results of the algorithms geNorm and NormFinder, best genes left. (a) Ranking by the geNorm algorithm; the graph shows the M-values for the different genes. (b) Ranking by the NormFinder algorithm; the graph shows the standard deviations (SD) for the different genes. (c) Accumulated standard deviations by NormFinder for different numbers of genes; the optimum is shown by a red indicator.

As mentioned already, the genes *SDH* and *RPS9* were excluded from the analysis of the *in planta* samples. Here geNorm identified *Elf1a* and *Elf3* as the best genes with *RPS14* third (Fig 3a). NormFinder calculated *Act* and *PDH* as best and *UbcE2* third. Two genes seem to be optimal for normalization (Fig 3b and 3c). For these stages the rankings are differing between the two algorithms. Only *Ubc* and *PDK* are consistently identified as the least stable genes. Since the values are all relatively low, the different rankings can be interpreted such as there seem to be small differences between the different genes and have similar suitability as reference genes.

**Fig 4:**
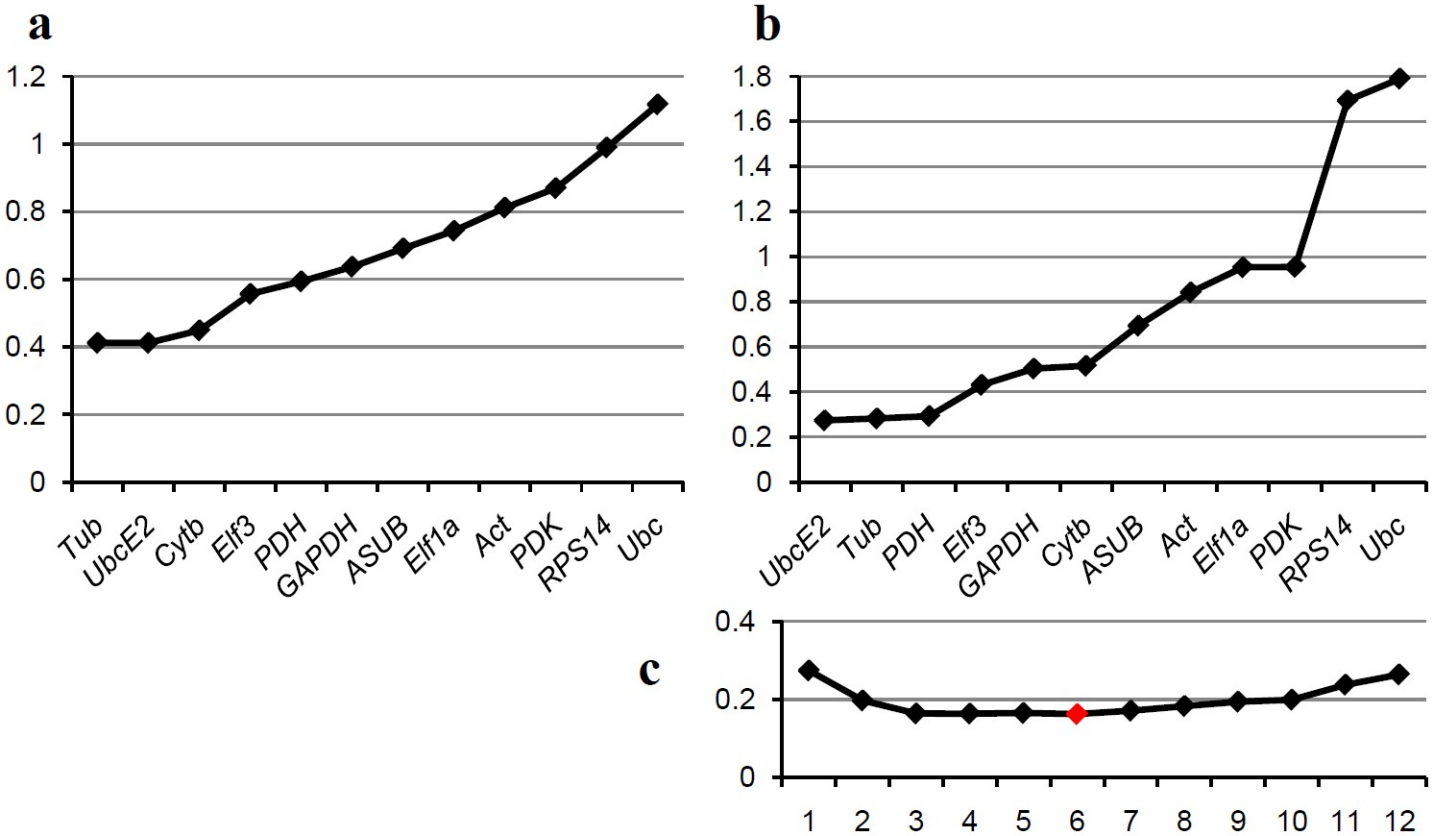
Comparative analysis of the candidate reference genes for *in vitro* and *in planta* samples. Genes are ranked based on the results of the algorithms geNorm and NormFinder, best genes left. (a) Ranking by the geNorm algorithm; the graph shows the M-values for the different genes. (b) Ranking by the NormFinder algorithm; the graph shows the standard deviations (SD) for the different genes. (c) Accumulated standard deviations by NormFinder for different numbers of genes; the optimum is shown by a red indicator.

An analysis of the genes over all stages (also without *SDH* and *RPS9*) resulted in *Tub* and *UbcE2* as best genes predicted by geNorm with *Cytb* as a third and *UbcE2*, *Tub*, and *PDH* as best with NormFinder (Fig 4a and 4b). For this analysis six reference genes are calculated as optimal (Fig 4). Since there is almost no difference in the accumulated standard deviation between the different combinations except between using only one reference gene or two, however, it seems that two reference genes should be sufficient again.

Optimally reference genes should be stably expressed and this should be reflected in a constant Cq or a narrow range of Cq values, when a constant amount of RNA/cDNA is used as template for PCR. Therefore, reference genes might be selected for the narrowest Cq range. There can, however, be reasons for changes in Cq that do not diminish the suitability of reference genes. Such reasons are the change from dormant spore to metabolically active germinating spore and the difference in measurement of only fungal RNA and a mixture of fungal and plant RNA. Since the algorithms geNorm and NormFinder consider only the differences between Cq values between the genes, these parallel shifts in Cq are irrelevant. To select reference genes that are both stable by the means of geNorm and Normfinder and also do not have a large Cq range, a combined score was used. For this, the rankings by the three parameters were added up and a new ranking was performed based on the sums (Table 3). In this overall ranking *SDH* was the best gene for *in vitro* samples, followed by *Elf3a* and *Tub*. For *in planta* samples it was *UbcE2*, followed by *PDH* and *Elf3a* together with *Cytb*. For all sampled stages *UbcE2*, *Elf3*, and *Tub* seem to be most suitable.

This study provides reference gene combinations for three sets of samples, *in vitro* stages, *in planta* stages and all samples together. Of these the all samples set is probably the most relevant, since studies, that aim to identify genes involved in the establishment and maintenance of the biotrophic interaction, i. e. effectors, are searching for genes that are up-regulated in *in planta* samples and have to compare *in vitro* and *in planta* samples. Studies using only the *in vitro* samples could be performed to specifically search for genes that are highly expressed in the appressorium while searches for genes that are involved in spore formation could be limited to the *in planta* stages.

The proposed reference genes are relatively similar for all three sets of samples, so the reference gene combinations can be exchanged. An exception is *SDH*, which should not be used for *in planta* samples and also *UbcE2* may not be the first choice for a study focusing entirely on the *in vitro* samples. Otherwise a combination of either two reference genes, *UbcE2* and *Tub* or three reference genes, *UbcE2*, *Tub*, and *Elf3* is recommended for normalization in RT-qPCR for gene expression profiling during the stages of urediospore infection of *U*. *appendiculatus* on *P*. *vulgaris*.

In our study for reference genes in *P*. *pachyrhizi* [17] we also included a sample infected with Bean Pod Mottle Virus (BPMV) to check the stability of the reference genes in a host induced gene silencing (HIGS) setup. Since we do not perform the corresponding experiments for *U*. *appendiculatus*, such a sample was not included here. Cooper et al. [13], however, could demonstrate the effectiveness of HIGS against *U*. *appendiculatus*. In their study they do not yet report measuring mRNA levels of the silenced gene during silencing. Based on our results for *P*. *pachyrhizi*, where no notable changes in Cq were observed for any of the candidate reference genes it can be assumed that the reference gene combinations that are best for either the *in planta* samples or all samples could also be used for assessing the success of HIGS experiments.

### Validation of the reference genes

To validate the reference gene combination *UbcE2*, *Elf3*, and *Tub*, these were tested for transcript profiling of effector candidates. We recently published a study identifying candidate effectors in *U*. *appendiculatus* [4]. Part of that study was to establish the expression patterns of these genes. For this analysis we used *Act* and *CytB* to normalize. This selection of reference genes was based on earlier results from this present study using the same strategy for selection of the best reference genes as presented above. In this earlier analysis the genes *GAPDH*, *PDK*, *RPS14*, and *Tub* were not included because at this time suitable primers for these genes had not yet been identified.

For testing the two effector candidates *Uaca_9* and *Uaca_12* that showed distinctive expression patterns in our earlier study were chosen. *Uaca_9* showed almost no changes in expression whereas *Uaca_12* showed strongly induced expression in the *in planta* samples.

**Fig 5:**
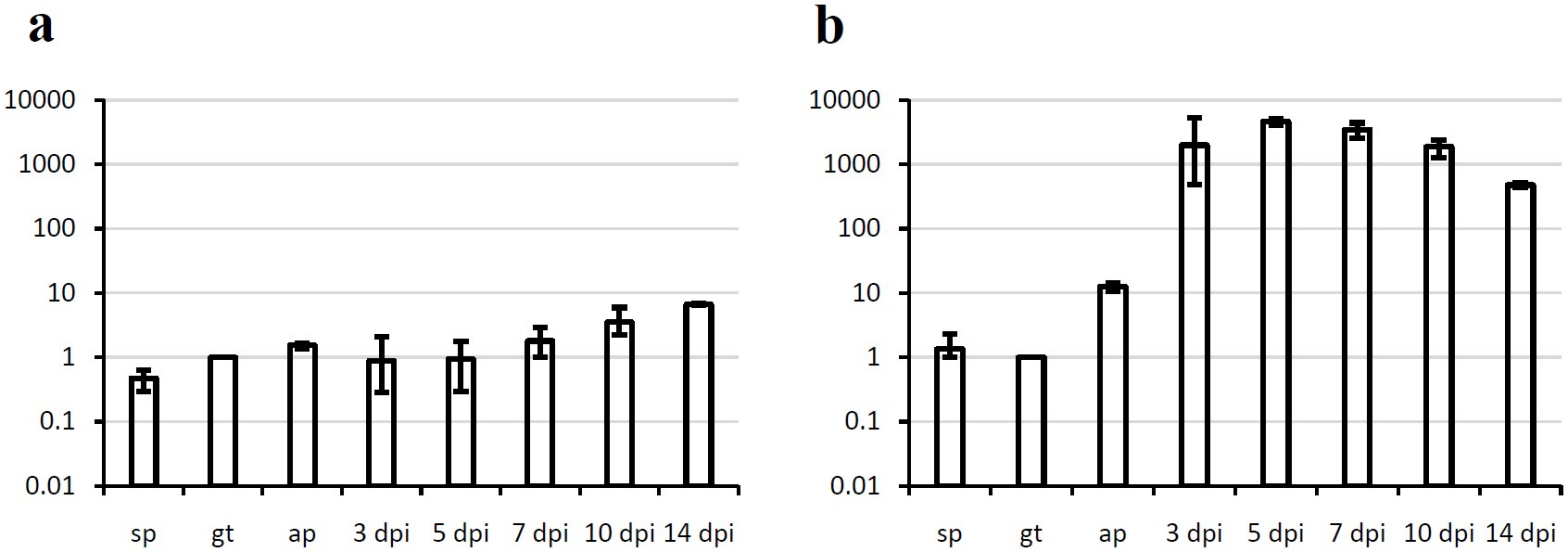
Expression patterns of two effector candidates. (a) *Uaca_9*. (b) *Uaca_12*. *UbcE2*, *Elf3*, and *Tub*, were used for normalization. Columns show the geometric means of three biological replicates; error bars indicate maximum and minimum values. All values are relative to the gt stage; ordinates show relative normalized expression.

In the experiment I now again found almost constant expression for *Uaca_9* and strong induction for *Uaca_12* (Fig 5). This can be accepted as additional evidence that the combination of reference genes *UbcE2*, *Elf3*, and *Tub* is suitable for normalization over all included samples. On the other hand, this analysis also validates the results of the old experiment. Even though in the final analysis *Act* and *CytB* were not among the best reference genes they are still among those that can be regarded as suitable for normalization. The similarity of expression patterns shown in Fig 5 and those in [4] corroborates this assumption. Therefore, it can be stated that suitable reference genes were identified and the expression patterns presented in [4] are valid.

## Conclusions

This study shows that Cq values of housekeeping genes vary between different samples representing the development of *U*. *appendiculatus* on *P*. *vulgaris*. Part of these variations is due to the fact that the *U*. *appendiculatus* RNA is either pure in the *in vitro* samples or mixed with *P*. *vulgaris* RNA in the *in planta* samples. But there are also definite changes in gene expression between the resting urediospore and the germ tube samples. Since the resting urediospore stage is metabolically inactive and differs in gene expression from all other stages it is not advisable to use this stage as a reference sample; the germ tube stage should be used instead as the sample relative to which expression should be measured.

The Cq values are relatively stable between gt and the ap sample. Compared to our results for *P*. *pachyrhizi* [17] this is a considerable improvement. It is, therefore, an important consideration to incubate the *in vitro* structures on PE sheets for 9 h to observe appressoria but not much longer.

Table 3 represents all rankings of the candidate reference genes. Using a combined ranking based on Cq ranges, geNorm, and NormFinder a combination of either two reference genes, *UbcE2* and *Tub* or three reference genes, *UbcE2*, *Tub*, and *Elf3* is recommended for normalization.

## Supporting information

S1 File Cq values_quartiles_figures. These tables contain the data underlying Fig 1. Calculations and graphs are also there. The same data were also used in the calculations with geNorm and NormFinder that led to Figs 2–4.
(XLSX)

S2 File Control non inoculated leaf. The table shows the raw Cq values from that experiment.
(XLSX)

S3 File Transcript profiling. The tables show all data underlying the transcript profiling experiment described with Fig 5.
(XLSX)
